# The Factors Influencing Children’s Helping Behavior: The Roles of Cognition and Empathy Concern

**DOI:** 10.3390/bs15050689

**Published:** 2025-05-16

**Authors:** Mingyue Liang, Hongfang Mo, Jipeng Duan

**Affiliations:** 1 Faculty of Teacher Education, Ningbo University, Ningbo 315211, China; liangmingyue@nbu.edu.cn (M.L.); 2211030004@nbu.edu.cn (H.M.); 2 Department of Psychology, Ningbo University, Ningbo 315211, China

**Keywords:** helping behavior, cognitive level, empathy concern level, behavioral level, children

## Abstract

Helping behavior plays an important role in children’s social interaction and personality development. This study used a situational test method to investigate the effects of cognition and empathy concern on helping behavior in children aged 6–12. The results revealed that empathy concern had a significant effect on children’s helping behavior compared to cognition. By inducing varying levels of empathy concern through prosocial songs, the subjects exhibited significant differences in helping behavior, with children in the high empathy concern group demonstrating significantly higher levels of helping behavior. Therefore, as for the education on children’s helping behavior, teachers should pay special attention to the factor of empathy concern, and the educational strategy of activating empathy concern can improve the effectiveness of children’s helping behavior education.

## 1. Introduction

Helping behavior, as a type of prosocial behavior (Eisenberg et al., 1995), is the behavior displayed by people who are willing to pay the costs of resources, time, and money to benefit others through a series of interactions (Weltzien et al., 2018; Eisenberg, 1986). Helping behavior has a significant impact on an individual’s interpersonal and social relationships (Laguna et al., 2020).

Recently, many studies have explored the internal mechanisms of these factors, mainly focusing on the two aspects of helping cognition and empathy concern. On the one hand, some scholars have emphasized that helping cognition has a greater influence on children’s helping behavior. Social cognitive theory emphasizes the role of cognitive initiation, which states that the behaviors individuals display depend on their cognitive thinking (Bandura, 1992). Therefore, individuals’ exposure to diverse external information influences their internal cognitive processing, subsequently impacting their social behaviors (Han et al., 2019). It has been found that children’s prosocial reasoning skills gradually increase with age (Chadha & Misra, 2006). It is not until adolescence that they are able to make prosocial decisions better and guide their behavior in a stable manner (Do et al., 2019). Children comply with social norms (Weltzien et al., 2018) and dynamically integrate social perceptions (e.g., whether helping is safe or norm-compliant) to calibrate their evaluations of prosocial actions (Marshall et al., 2023). And children gradually internalize and develop self-values and acquire problem-solving skills during the socialization process, which guides the implementation of individual helping behaviors. Studies have found that children who value self-transcendence over self-improvement are more helpful than other children (Misgav et al., 2023); children’s judgments and assessments of their own abilities, the goals and avenues of helping, and the nature of the content of the programs received by the recipients and assessments affect their helping behavior (Bridgers & Gweon, 2018; Regan & Gutierrez, 2005). Research has found that when helping becomes common knowledge of the group, preschoolers become more motivated to fulfill their obligations, and more helping behaviors are shown (Siposova et al., 2021). Scholars who emphasize cognitive influences often place more emphasis on “direct education” to promote children’s helping behaviors. For example, by designing a one-year training course on mutual help, children continued to exhibit helping behaviors even after the formal intervention ended (Gillies, 2000). Children exhibit more helping behavior when their mothers establish helping as an obligatory perception (Giner Torréns & Kärtner, 2017; Fonseca et al., 2018). Children who were exposed to prosocial television news were more likely to engage in helping behaviors compared to those in the control group (De Leeuw et al., 2015); children who played prosocial video games showed significantly higher levels of helping behavior compared to those in the control conditions (Greitemeyer & Osswald, 2010).

On the other hand, some scholars have emphasized that empathy concern has a greater impact on children’s helping behaviors. Empathy concern, the other-centered, consistent emotion that arises through witnessing another person’s suffering, includes emotions such as sympathy and soft heartedness (C. D. Batson et al., 1987). Different scholars have each focused on the content of empathy and developed theoretical explanations. Hoffman (2000) focuses on the development of empathy in children’s growth process, and believes that individuals go through four developmental stages of empathy from infancy to late childhood, including Global Empathy, Egocentric Empathy, Empathy for Another’s Feelings, and Empathy for Another’s Life Condition. In the process, children develop the ability to recognize others’ emotions and empathize with their suffering. With cognitive maturation, children’s empathy extends beyond immediate, salient victims to abstract or generalized others (e.g., “the poor” or “people in war”). This shift reflects an advanced capacity to represent and respond to collective suffering. Eisenberg and Fabes (1990) divided empathy into affective empathy and cognitive empathy. The former refers to the individual’s automatic emotional response to the emotional states of others. The latter refers to the individual’s ability to understand others’ emotional and psychological states. The two can be common to the individual’s prosocial behavior, but also can be developed independently. Tousignant et al. (2017) have noted that emotional empathy emerges before cognitive empathy in early childhood. Decety and Cowell (2014) focus on neuroscientific models of empathy, including the mirror neuron system for Affective Sharing, the right temporoparietal joint area of the brain for Self–Other Awareness, and the prefrontal cortex for Top-Down Regulation. It is believed that empathy may trigger altruistic behavior, but also lead to prejudice, which requires cognitive regulation. Therefore, childhood is an important stage in the development of an individual’s empathic ability. At the same time, the relationship between empathy and cognition, which is linked to regulation or independent development, provides room for research on the factors influencing individual prosocial behavior. It has been found that empathy concern is related to personal traits, this type of empathy concern is more stable within an individual, and those who embody it will have a greater willingness to help others (Xiao et al., 2021; Bohns & Flynn, 2021). Scholars who emphasize the influence of empathy concern often place more emphasis on “environmental shaping” to promote children’s helping behaviors. Environmental factors such as prosocial content, the degree of identity similarity, and access to recipient information can trigger empathy concern in individuals, thereby promoting their helping behavior. Studies have found that prosocial media (TV programs and games) can induce empathy concern and promote prosocial behaviors in children (Prot et al., 2014; Shoshani et al., 2022; Harrington & O’Connell, 2016). When recipients imitate the behavior of helpers, it can trigger helpers’ empathy concern, leading to increased helping behavior and offsetting the psychological burden of perceiving a helping cost (Müller et al., 2012). Shared identity concepts within a group can trigger individuals’ empathy concern and promote their helping behavior (Miyazono & Inarimori, 2021). The more effort recipients make to overcome their difficulties, the more it can evoke empathy concern and helping behavior (Jin et al., 2021). In the development of children’s helping behaviors, educators must clarify the difference between cognitive and empathic concern influences on it. Based on this, targeted educational strategies can be designed to enhance the effectiveness of moral education.

Previous studies have mostly used questionnaires and experimental methods to investigate experimental variables, but questionnaires suffer from a number of problems: they lack a focus on the same structure and dimensions to be measured and the overall convergence and correlation between items are relatively low, which can reduce the interpretability and replicability of self-reported feelings (Desmedt et al., 2022). Moreover, experimental methods also have the problem of being disconnected from real-life scenarios (Treviño et al., 2014). Therefore, in this study, considering the above issues, the field experimental method was chosen to complete the study, in which the authors observed the subjects’ performance of the given task and evaluated them by telling a story that is closely related to the subjects’ lives.

All subjects in this study were selected from the nine-year school in China, located in an urban area, and all subjects were from Zhejiang Province or nearby provinces. The reasons for choosing children in this age group as subjects are twofold: On the one hand, the helping behavior displayed by children in this age group is vulnerable to interference and the diversified development of external factors (Martin & Olson, 2015), and this age group is in a critical period of helping behavior development, which has attracted the attention of scholars. On the other hand, the level of thinking of children in this age group is in the stage of Piaget’s development of formal operations, where children’s ability to understand concepts increases. Therefore, it is possible for children of this age to cooperate with the authors to conduct experimental research. The resulting self-reported data can reduce the experimental error to a certain extent and improve the authenticity of the experiment. After obtaining permission from the subjects themselves and their parents, the authors carried out the experiment; at the end of the experiment, the authors explained the purpose of the experiment to the subjects. The participation of the subjects in this study was approved by the Institutional Review Board (IRB) of Ningbo University.

## 2. Study 1: The Roles of Cognition and Empathy Concern in Children’s Helping Behavior

### 2.1. Participants

To be conservative and ensure that the results were reliable, a medium conventional effect size (f = 0.25) was planned for this experiment. A power analysis using G*Power 3.1 (Faul et al., 2009), with an alpha level of 0.05 and a power of 0.95, indicated that a minimum effect size of 70 was needed in each between-subjects condition. Therefore, we recruited 90 valid participants. All participants provided written informed consent for participation.

### 2.2. Experimental Design and Procedure

#### 2.2.1. Experimental Design

This study used a one-way between-subjects design, with the independent variables being the level of helping cognition and the level of empathy concern exhibited by children aged 6–12 years in the context test and the dependent variable being the children’s level of helping behavior.

##### Test Materials for Cognitive Level

The authors prepared 5 pictures with helping behavior content and 5 pictures with non-helping behavior content and used E-Prime 2.0 software to input and display the 10 pictures, with the number of pixels in the pictures set to 667 × 500. The picture playback was set to an infinite duration where the subjects answered the questions about one picture before moving to the next picture. The subjects were required to judge whether the content of the pictures depicted helping behavior. In accordance with the standard, they pressed the N or M key, and one point for correct judgment (10 points total).

##### Test Materials for Empathy Concern Level

The authors used a situational test method in which the subjects were presented with a situational story of a mother and daughter who were homeless and hungry in a snowy winter, and the subjects reported their feelings related to the content on a scale after reading the story. The scale was developed using the State Empathic Response Scale developed by C. D. Batson et al. (1995), and five words describing emotions (unfortunate, miserable, pitiful, sympathetic, and compassionate) were selected based on J. G. Batson et al.’s (1991) empathy–altruism hypothesis, which uses a five-point scale (1 for “not at all” and 5 for “full agreement”). All the subjects were asked to rate the five emotions they felt, with higher scores indicating greater empathy concern^1^.

##### The Materials for Testing Helping Behavior Level

The authors used the situation test method; the test point is whether the subjects are still willing to make the choice to help others even though they know that they need to pay the costs of time and money. Two related scenarios were designed. In scenario 1, the subjects were asked to give up their weekend or rest time to assist the class teacher in setting up the classroom for the parent–teacher conference the following week, and in scenario 2, the subjects were asked to donate the money saved to buy a long-desired toy for someone in need. The subjects made their choices after reading the story scenarios, and their level of helping behavior was measured on a five-point scale (1 for “spending no time/money to help” and 5 for “spending all the time/money to help”), with higher scores indicating higher levels of helping behavior.

### 2.3. Experimental Procedure

After the subjects entered the experimental site, they were first required to complete the cognitive level test. The specific operation process was as follows: the authors provided a detailed explanation of the operation rules. Then, the authors ensured that the subjects understood, and after receiving a positive response from the participants, they were allowed to independently begin the process. The E-prime 2.0 software first played the welcome speech, and then the subjects indicated that they were ready to start the process by pressing the space bar on the computer. The E-prime 2.0 software interface then displayed a gaze point lasting 1000 ms and 10 pictures in a chaotic sequence, and the subjects judged whether the content of the pictures displayed helping behavior by pressing the N or M key when they were ready.

The subjects were then instructed by the authors to complete a scenario test on the level of empathy concern and helping behavior. Upon completion, the authors informed the subjects that they had completed the process and paid them for the experiment.

### 2.4. Results

The authors used linear regression analysis to explore the relationship between helping cognition, empathy concern, and children’s helping behavior. The authors grouped the participants’ helping cognitive and empathy concern scores into high and low groups based on a 27% scale (Kelley, 1939)^2^ and categorized the participants’ helping behavior performance scores under different cognitive and empathy concern levels. In addition, to further illustrate the effects of cognitive and empathy concern levels on helping behaviors and to explore the relationships among the three factors, structural equation modeling was also conducted in this project.

Linear regression was used to analyze the relationship between children’s helping cognition, empathy concern level, and helping behavior, and as can be seen in Table 1, children’s empathy concern level was a significant predictor of helping behavior (*β* = 0.497, *** *p* < 0.001; *β* = 0.623, *** *p* < 0.001); children’s helping cognition level was not a significant predictor of helping behavior (*β* = −0.010, *p* = 0.868; *β* = −0.064, *p* = 0.326).

#### 2.4.1. Effects of Different Levels of Helping Cognition on Subjects’ Helping Behavior

Using the 27% proportionality criterion for grouping, it was determined that the lowest critical score for the high cognitive level group was 9 out of 10, totaling 45 people; the highest critical score for the low cognitive level group was 7 out of 10, totaling 31 people. Data analysis using an independent-samples *t*-test showed that the difference in the level of helping behavior requiring a monetary cost across cognitive levels was not significant, *t*_(74)_ = −0.634, *p* > 0.05, Cohen’s d = −0.140, 95% CI = [−0.64, 0.33]. The difference in the level of helping that required a time cost across cognitive levels was not significant, *t*_(74)_ = 0.565, *p* > 0.05, Cohen’s d = 0.128, 95% CI = [−0.40, 0.71]. Therefore, the effect of helping cognition on subjects’ helping behavior was not significant.

#### 2.4.2. The Effect of Different Levels of Empathy Concern on Subjects’ Helping Behavior

**Figure 1:**
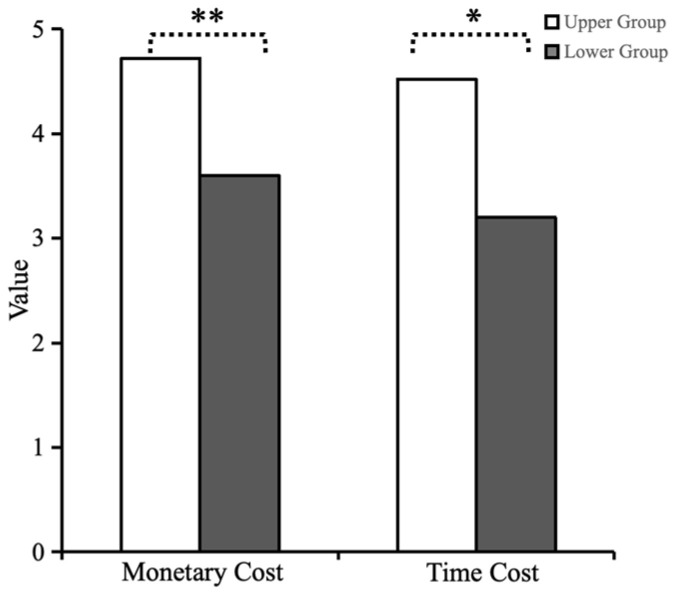
Differences in the mean values of helping behavior between the high and low empathy concern groups. Note: Asterisks denote statistical significance: * *p* < 0.05, ** *p* < 0.01.

Using the 27% proportion criterion for grouping, it was determined that the lowest critical score for the high empathy concern ability level grouping was 5 points (out of 5), totaling 25 people; the highest critical value for the low empathy concern ability level grouping was 3.6 points (out of 5), totaling 30 people. Data analysis using an independent-samples *t*-test showed that the level of helping behavior of subjects with high levels of empathy concern competence in the condition involving the need to pay a monetary cost (*M* = 4.72, *SD* = 0.614) was significantly greater than the level of helping behavior of subjects with low levels of empathy concern competence (*M* = 3.60, *SD* = 1.133), *t*_(53)_ = −4.428, ** *p* < 0.01, Cohen’s d = −0.989, 95% CI = [−1.63, −0.62]. The level of helping behavior of subjects with high levels of empathy concern (*M* = 4.52, *SD* = 0.918) was significantly greater than that of subjects with low levels of empathy concern (*M* = 3.20, *SD* = 1.243) in the condition involving the need to incur a cost of time, *t*_(53)_ = −4.400, * *p* < 0.05, Cohen’s d = −1.061,95% CI = [−1.92, −0.72]. Thus, empathy concern had a significant effect on subjects’ helping behavior (Figure 1).

#### 2.4.3. Structural Equation Modeling Results

**Figure 2:**
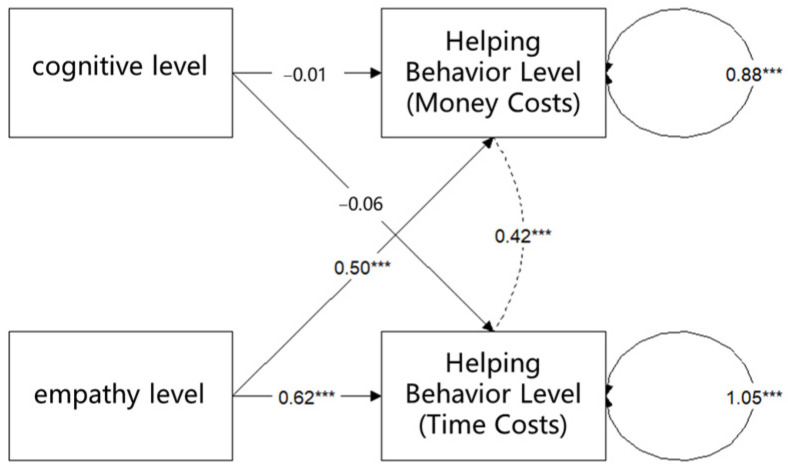
Structural equation modeling fitting results for study 1. Note: Asterisks denote statistical significance: *** *p* < 0.001.

The level of empathy concern and the level of cognition were used to predict the two levels of helping behavior (monetary cost and time cost), and structural equation modeling was conducted. The model was estimated using the maximum likelihood (ML) method. To assess model fit, the following indices were reported: comparative fit index (CFI), Tucker–Lewis index (TLI), root mean square error of approximation (RMSEA), and standardized root mean square residual (SRMR). According to the recommendation of Kline (2011), a well-fitted model should satisfy the following conditions: the CFI and TLI values are greater than 0.95, and the RMSEA and SRMR values are less than 0.06. According to these criteria, the current model fits the data well, χ^2^ (2) = 1.097, *p* = 0.578, CFI = 1.000, TLI = 1.054, RMSEA = 0.000, and SRMR = 0.030. As shown in Figure 2 and Table 2, there was no significant relationship between the level of cognition and either the level of helping behavior requiring a monetary cost (*b* = −0.01, *p* = 0.865) or the level of helping behavior requiring a time cost (*b* = −0.06, *p* = 0.965); the level of empathy concern was significantly related to both the level of helping behavior requiring a monetary cost (*b* = 0.50, *** *p* < 0.001) and the level of helping behavior requiring a time cost (*b* = 0.62, *** *p* < 0.001). This result again suggested that empathy concern has a significant effect on helping behavior.

## 3. Study 2: The Effect of Empathy Concern Level on Children’s Helping Behavior Level

### 3.1. Subjects

Study 1 revealed a significant correlation between empathy concern and children’s helping behavior, and study 2 focused on examining whether children’s levels of helping behavior changed under conditions that initiated different levels of empathy concern in the children, aiming to explore causal relationships and offer insights for practical interventions.

Studies have shown that the use of musical interventions can improve empathy concern in children; for example, Kalliopuska and Ruokonen (1993) designed a holistic empathy concern education program involving musical exercises that spanned more than three months and reported that the level of empathy concern and prosociality of the children who participated significantly increased. Schellenberg et al. (2015), who designed a ten-month group music training program, reported that the children who participated experienced increases in empathy concern and prosocial behavior. Greitemeyer (2009) concluded that playing prosocial songs can increase individuals’ ability to empathize and promote prosocial behavior.

One week later, the authors randomly divided the subjects into 2 groups, with the experimental group administered the prosocial song (48% boys, 52% girls; *M* = 7.92, *SD* = 1.52) and the control group administered the neutral song (57% boys, 43% girls; *M* = 7.90, *SD* = 1.48). The authors used linear regression analysis to examine the relationship between perceptions of helpfulness, empathy concern, and children’s helpful behavior. To examine the effects of different levels of empathy concern on subjects’ helping behaviors, the authors grouped the participants based on their empathy concern scores as high or low based on a 27% scale (Forlano & Printer, 1941) and compared subjects’ helping behavior performance at the different levels of empathy concern. Moreover, to compare and further confirm the relationships among cognitive level, empathy concern level, and the two levels of helping behavior, the same structural equation modeling analysis was conducted in this experiment.

### 3.2. Experimental Design and Procedure

#### 3.2.1. Experimental Design

This study utilized a one-way within-subjects design, with the independent variable being the level of empathy concern, the dependent variable being the child’s level of helping behavior, and the operative condition being the imposition of a prosocial song.

##### Prosocial Songs

The authors chose eight songs from the library based on the dimensions of prosocial lyrics contained in the songs, popularity, etc., namely, For Future Self, Shaonian, Guangliang, Childhood, Contentment, As I Wish, Together Toward the Future, and Embracing You, and then randomly chose 20 children in grades 1–6 who were not to be involved in the experiment, and invited them to listen to two of the songs. After listening to the songs, children completed questionnaires. The questionnaire consisted of three parts: the Song Rating Questionnaire (Anderson et al., 2003), the Positive and Negative Sentiment Scale (PANS), and the Perceived Arousal Scale (PAS, Anderson et al., 1995). A 5-point scale was used to assess the song’s prosociality, favorability, and arousal perception (with 1 denoting “not very much” and 5 “very much”), and two prosocial songs and two neutral songs were selected to be played randomly in the experiment.

A one-sample *t*-test revealed a significant difference in the means of the eight songs (*t*_(7)_ = 15.128, ** *p* < 0.01). The two songs with the highest prosocial scores, namely, As I Wish (*M* = 4.6, *SD* = 0.89) and Embracing You (*M* = 4.4, *SD* = 0.55), were chosen as prosocial randomized songs. The two with the lowest scores, Childhood (*M* = 2.8, *SD* = 1.30) and For My Future Self (*M* = 2.8, *SD* = 1.30), served as neutral randomized songs. In addition, the difference between the means of the two groups of songs was not significant in the content survey about favoritism and arousal^3^.

##### Test Materials for Empathy Concern, Cognitive Level, and Helping Behavior Level

The authors used the situational test method to present the subjects with a situational story about a peer, Xiaoming, whose family lived in a remote mountainous area and had poor learning and living conditions. All subjects reported their feelings related to the content on the scale after reading the story. The scale for empathy concern test was the same as in study 1.

The authors prepared 5 pictures with helping behavior content and 5 pictures with non-helping behavior content. The authors changed the contents of the photos to reduce the experimental impact of repetition effects and the procedure of cognitive level test was the same as in study 1.

The authors used a situational test, and the test point was whether the subjects were still willing to make the choice to help others even though they knew that it would cost them time and money. Two related scenarios were designed: scenario 1 concerned whether subjects were willing to give up their weekend or rest time to cooperate with the experimenter (teacher) in completing a survey, and scenario 2 concerned whether subjects were willing to donate the money saved to purchase a long-desired toy for a person in need. The subjects made their choices after reading the story scenarios, and their level of helping behavior was measured on a five-point scale (1 for “no time/money spent helping” and 5 for “all time/money spent helping”), with higher scores indicating higher levels of helping behavior.

#### 3.2.2. Experimental Procedure

After the subjects entered the experimental place, the authors informed the subjects that a song would be played next and asked them to listen carefully. Then, the authors randomly played one of the prosocial songs. After the subjects finished listening to the song, the authors instructed the subjects to complete the situational test on the level of empathy concern, cognitive level, and helping behavior. Finally, the authors informed the subjects that they had completed the process and gave them payment for participating in the experiment (see Supplementary Materials for the specific operation process).

### 3.3. Results

#### 3.3.1. Effects of Different Types of Songs on Children’s Empathy Concern Levels

Data were analyzed using paired-samples *t*-tests, and empathy concern level pre- and post-tests were conducted separately for subjects with prosocial and neutral song manipulations implemented. The results showed that the difference between the pre- and post-tests of empathy concern levels of the subjects who were administered the neutral song manipulation was not significant, whereas in the data analysis of empathy concern levels of the subjects who were administered the prosocial song manipulation, the subjects who were in the group with low empathy concern levels in the pre-tests (16) showed a significant increase in their empathy concern, and the post-tests’ empathy concern levels (*M* = 3.88, *SD* = 1.02) were significantly higher than the pre-tests’ empathy concern levels (*M* = 3.05, *SD* = 0.60), and *t*_(15)_ = −2.948, * *p* < 0.05, Cohen’s d = −0.810, 95% CI = [−1.42, −0.22]. Thus, the prosocial songs had a significant effect on the initiation of empathy concern levels in the low empathy concern level group of the experimental group.

#### 3.3.2. Effects of Different Levels of Empathy Concern on Children’s Helping Behavior

Using linear regression to analyze the relationship between children’s helping cognition, level of empathy concern, and helping behavior, Table 3 shows that children’s level of empathy concern was a significant predictor of helping behavior (*β* = 0.389, * *p* = 0.030; *β* = 0.515, ** *p* = 0.004); children’s level of helping cognition was not a significant predictor of helping behavior (*β* = 0.056, *p* = 0.531; *β* = 0.011, *p* = 0.896).

**Figure 3:**
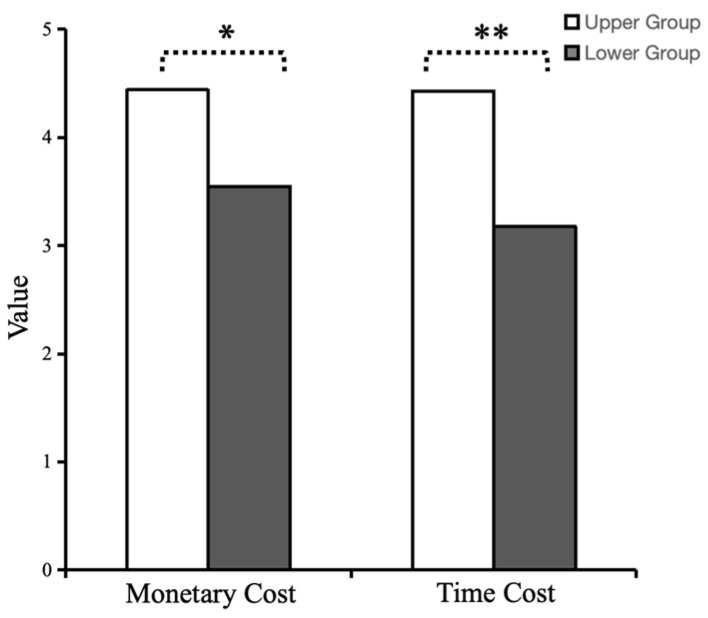
Differences in the mean values of helping behavior between the high and low empathy concern groups. Note: Asterisks denote statistical significance: * *p* < 0.05, ** *p* < 0.01.

The concern level scores of the subjects in the experimental group were grouped on a 27% scale criterion (Kelley, 1939); the lowest critical value for the high empathy concern competence level group was 5 points (out of 5), totaling 18 people, and the highest critical value for the low empathy concern competence level group was 3.2 points (out of 5), totaling 11 people. Data analysis using an independent-samples *t*-test showed that the level of helping behavior of subjects with high levels of empathy concern who needed to pay a monetary cost (*M* = 4.44, *SD* = 0.784) was significantly greater than that of subjects with low levels of empathy concern (*M* = 3.55, *SD* = 1.128), *t*_(27)_ = −2.536, * *p* < 0.05, Cohen’s d = −0.797, 95% CI = [−1.63, −0.17]. The level of helping behavior of subjects with high levels of empathy concern (*M* = 4.33, *SD* = 1.029) was significantly greater than that of subjects with low levels of empathy concern (*M* = 3.18, *SD* = 0.982) under the condition that the subject would incur a cost of time, *t*_(27)_ = −2.974, ** *p* < 0.01, Cohen’s d = −1.120, 95% CI = [−1.95, −0.36]. Meanwhile, an independent-samples *t*-test of the cognitive levels corresponding to subjects with high and low levels of empathy concern revealed that there was no significant difference between the cognitive levels of the two groups, *t*_(27)_ = −1.01, *p* = 0.319, Cohen’s d = −0.36, 95% CI = [−1.99, 0.67]. Thus, the effect of empathy concern initiated by the subjects in the prosocial song condition on helping behavior was significant (Figure 3).

#### 3.3.3. Structural Equation Modeling Results

**Figure 4:**
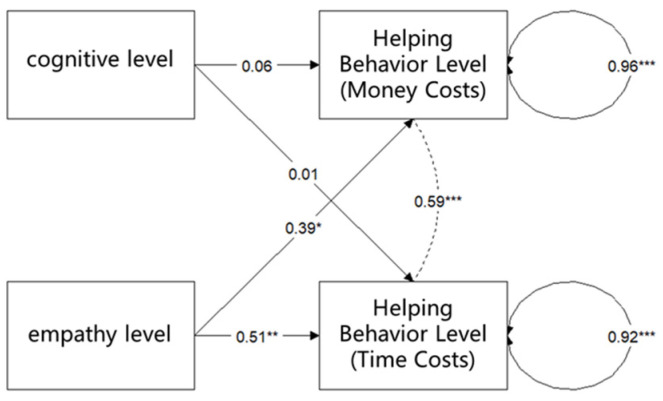
Structural equation modeling fitting results for study 2. Note: Asterisks denote statistical significance: * *p* < 0.05, ** *p* < 0.01, *** *p* < 0.001.

As in study 1, the level of empathy concern and the level of cognition were used to predict the two levels of helping behavior (monetary cost and time cost), and structural equation modeling was conducted. The model was also estimated using the maximum likelihood (ML) method. To assess the model fit, the following indices were also reported: comparative fit index (CFI), Tucker–Lewis index (TLI), root mean square error of approximation (RMSEA), and standardized root mean square residual (SRMR). According to these criterions, the current model fits the data well, χ^2^ (2) = 0.554, *p* = 0.758, CFI = 1.000, TLI = 1.140, RMSEA = 0.000, and SRMR = 0.030. As shown in Figure 4 and Table 4, there was no significant difference between the level of cognition and the level of helping behavior that entails a monetary cost (*b* = 0.06, *p* = 0.511), and the level of helping behavior that entails a time cost (*b* = 0.01, *p* = 0.891) was not significantly related to the level of cognition. There was a significant relationship between the level of empathy concern and both the level of helping behavior that required a monetary cost (*b* = 0.39, * *p* = 0.020) and the level of helping behavior that required a time cost (*b* = 0.51, ** *p* = 0.002). This result again suggested that empathy concern has a significant effect on helping behavior.

## 4. Discussion

This study used situational testing and found that in study 1, cognition does not play a significant role in children’s helping behavior, empathy concern plays a significant role in children’s helping behavior, there is a significant difference in the performance of children’s helping behavior with different levels of empathy concern, and empathy concern plays a significant role in the prediction of helping behavior.

On this basis, in study 2, the authors imposed the experimental manipulation of prosocial songs and neutral songs on the subjects to test the initiating effect of different types of songs on the empathy concern level and the differences in the performance of the subjects’ helping behavior under the initiation of different empathy concern levels. It was found that prosocial songs had a significant effect on the initiation of children’s empathy concern level, and the empathy concern level of the group of children with low subgroups in the experimental group on the pre-test was significantly enhanced; the differences in the performance of children’s helping behaviors in children initiated with different levels of empathy concern were significant, and empathy concern had a significant role in the prediction of the helping behaviors, which is in line with the conclusions of study 1.

Existing research has not yet reached a unified conclusion on the role of cognition and empathy concern on children’s helping behavior. In this study, it was found that there is a difference between the effects of empathy concern and cognition on children’s helping behavior, with empathy concern playing a more significant role than cognition. Regarding this difference, the authors propose the following reasons. On the one hand, it has been found that children are able to use strategic means to help themselves achieve potential goals (Grueneisen & Warneken, 2022), causing the nature of the behavior to change. The cognitive complexity created by growth and the educational environment (Flook et al., 2019) leads to a fragile state of cognitive initiation in children, which is prone to turn into selfish bias (Weltzien et al., 2018) and somewhat reduces the likelihood that children will engage in prosocial behavior. To address the above problems, current studies propose leveraging normative influences, where the power of norms is utilized. Substantial changes in prosocial behavior may be due to its combination with children’s understanding of norms (House & Tomasello, 2018), and guiding children to label prosocial behaviors as “norms” can help children identify with and act on prosocial concepts. On the other hand, empathy concern, a moral emotion, is a force that cannot be ignored by individuals. It has been found that human beings exhibit helping behaviors at an early age and that these behaviors are spontaneous and purely altruistic (Warneken & Tomasello, 2013; Hepach et al., 2012). Individuals are inherently equipped with the ability to pay attention to and perceive the emotions of others, as well as the motivation to make efforts that benefit improving the circumstances of others, which is similar to the expression of empathic abilities. Inducing empathy concern in children can help individuals overcome cognitive limitations and facilitate the occurrence of helping behaviors (Sierksma et al., 2015), which is also consistent with the findings of this study. Based on sympathy, prosocial behavior can balance individual reciprocity and selfish motives, prompting individuals to pursue altruistic motives (Grueneisen & Warneken, 2022), thereby fostering children’s prosocial behavior. Therefore, the empathy concern factor plays an important role in the formation of both good helping cognition and behavior. To develop helping behavior in children at this stage, educators should pay attention to children’s emotional factors and initiate empathy concern through appropriate teaching measures to promote their understanding of the teaching content and the development of their own helping behavior.

This study provides valuable insights into the role of empathy in children’s helping behavior, but several limitations need to be considered. First, the sample size was relatively small, limiting the generalizability and statistical power of the findings. Future research should aim to recruit a larger and more diverse sample to test the replicability and ecological validity of the results. Second, this study focused on specific aspects of cognition and empathy concern but lacked a clear conceptual differentiation between similar or distinct categories. Further exploration is needed to clarify the relationships between these concepts and how different types of cognition and empathy concern influence helping behavior. Additionally, this study did not use widely recognized and well-validated empathy scales, which may affect the accuracy and consistency of the findings. Future studies should utilize more reliable and effective measures of empathy to better capture its role in prosocial behaviors. Finally, the participants in this study were all from China, and the cultural values of the participants were influenced by collectivism, which may affect the cultural generalizability of the findings to a certain extent. Future research should further validate the generalizability of the findings in other cultural contexts, as well as take into account the issue of cultural equivalence in the selection of research instruments.

## Figures and Tables

**Table 1:** Linear regression test of the role of cognition and empathy concern in predicting children’s helping behavior.

Linear Regression Equation		Overall Fit Index			Significance of Regression Coefficients		
Outcome Variables	Predictor Variables	*R*	*R* ^2^	*F*	*β*	*t*	*p*
Helping Behavior (Money)	Empathy Concern Level	0.399	0.159	8.243	0.497	4.055	<0.001 ***
	Cognition Level				−0.010	−0.167	0.868
Helping Behavior (Time)	Empathy Concern Level	0.449	0.202	11.001	0.623	4.657	<0.001 ***
	Cognition Level				−0.064	−0.988	0.326

Note: Asterisks denote statistical significance: *** *p* < 0.001.

**Table 2:** The fit index of structure model in study 1.

χ^2^	*df*	*p*	RMSEA	SRMR	CFI	TLI
1.097	2	0.578	0.000	0.030	1.000	1.054

**Table 3:** Linear regression tests of the predictive effects of cognition and empathy concern on children’s helping behavior.

Linear Regression Equation		Overall Fit Index			Significance of Regression Coefficients		
Outcome Variables	Predictor variables	*R*	*R* ^2^	*F*	*β*	*t*	*p*
Helping Behavior (Money)	Empathy Concern Level	0.363	0.132	2.968	0.389	2.248	0.030 *
	Cognition Level				0.056	0.633	0.531
Helping Behavior (Time)	Empathy Concern Level	0.443	0.197	4.771	0.515	3.041	0.004 **
	Cognition Level				0.011	0.132	0.896

Note: Asterisks denote statistical significance: * *p* < 0.05, ** *p* < 0.01.

**Table 4:** The fit index of the structure model in study 2.

χ^2^	*df*	*p*	RMSEA	SRMR	CFI	TLI
0.554	2	0.758	0.000	0.030	1.000	1.140

## Data Availability

The data that support the findings of this study are available from the authors upon reasonable request.

## Notes

1 The Empathy Concern Scale (ECS) developed by C. D. Batson et al. (1987) consists of two main components, the Situation and the Empathy Scale test. The empathy–altruism hypothesis (J. G. Batson et al., 1991) found that compared to warm, alarmed, and perturbed, all of the empathy, distress, and sadness adjectives had higher scores on the component analysis (with varimax rotation) of responses to the adjectives. This study builds on this by selecting five adjectives of this type to test the empathic responses of the subjects. This scale has also been used in subsequent studies, for example, Greitemeyer’s (2009) test of state empathy. Based on the operational validity of the existing research, this scale was selected and used in this study to complete an experimental measure of the subjects’ level of empathy.

2 Kelley (1939) suggested when the scores are normally distributed, the upper and lower group is an effective method of distribution that allows both the greatest possible difference between the two comparison groups and the largest possible number of people in both groups. When the validity scale scores are flatter than the normal distribution, the ratio of the high grouping to the low grouping should be slightly higher than 27%, at around 33%. In general, a ratio between 25% and 33% is sufficient. However, in the case of standardized tests, it is still customary to use 27%. The authors ranked the scores of the variables used for grouping in descending order and determined the number of subjects by taking the total number of subjects * 27% and counting 27% of the subjects if they had the same score.

3 The duration of the songs selected for the experiment was controlled at 3–4 min, and there were two prosocial songs and two non-prosocial songs in total. After the subjects were grouped together, one of the two songs was randomly selected for the intervention. A brief introduction of the four songs is as follows: 1. As I Wish: a delicate depiction of the dedication and expectation of the fathers and the lyrics of the song, *And I’ll love the earth you love*, convey gratitude and inheritance for this guardianship. 2. Embrace You: a healing song about loneliness and warmth. Through the simple but powerful action imagery of embrace, it sings a tender promise. 3. Childhood: the song recreates the innocence and happiness of that smartphone-less era in a descriptive way. 4. To the Future Self: the song tells in the first-person tone of a person’s journey to rediscover his courage after experiencing setbacks.

## Supplementary Materials

The following supporting information can be downloaded at https://www.mdpi.com/article/10.3390/bs15050689/s1.
