# Supplementary figures and images for: The Factors Influencing Children’s Helping Behavior: The Roles of Cognition and Empathy Concern
Source: Behav Sci (Basel). 2025 May 16;15(5):689. doi: 10.3390/bs15050689 (PMC12108753; doi:10.3390/bs15050689)

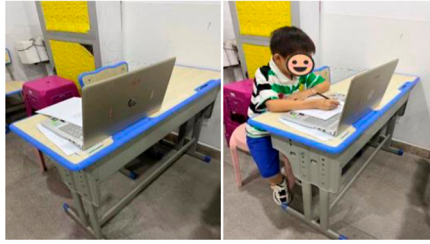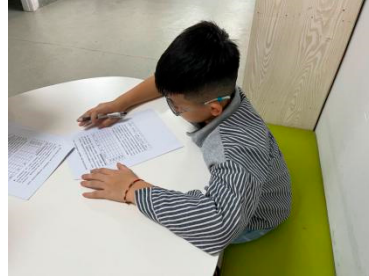

Supplementary figure S1. Operation of the experiment (3.2.2 Experimental Procedure)

Supplement: Supplementary file 1 [file behavsci-15-00689-s001.zip › behavsci-3535744-supplementary.pdf]
